# Surgical wound fluids from patients treated with intraoperative radiotherapy induce radiobiological response in breast cancer cells

**DOI:** 10.1007/s12032-018-1243-z

**Published:** 2018-12-31

**Authors:** Igor Piotrowski, Katarzyna Kulcenty, Dawid Murawa, Wiktoria Suchorska

**Affiliations:** 1 Radiobiology Laboratory, Greater Poland Cancer Centre, Garbary 15 Street, 61-866 Poznan, Poland; 2 Department of Electroradiology, Poznań University of Medical Sciences, Garbary 15 Street, 61-866 Poznan, Poland; 3 Department of General and Minimally Invasive Surgery, Poland Baptism Monument Hospital, 3 Maja 37, 62-200 Gniezno, Poland

**Keywords:** Breast cancer, Intraoperative radiotherapy, Radiation-induced bystander effect, Wound healing, DNA damage

## Abstract

Breast cancer is the most common cancer occurring in women. The standard of breast cancer treatment is based on breast-conserving surgery with administration of adjuvant whole breast radiotherapy. Research shows that in-breast relapse is most likely to occur in the tumour bed, i.e. around the scar. Intraoperative radiotherapy (IORT), in which radiation is delivered to the tumour bed, reduces the risk of local recurrence not only through direct cell killing, but also through modification of local microenvironment. Additionally IORT modifies the composition and biological activity of surgical wound fluid. Since many researchers show that radiation damage is mediated through factors secreted to the environment by irradiated cells, we hypothesized that this radiation-induced bystander effect is partly responsible for the change observed in surgical wound fluids. We collected conditioned medium from irradiated breast cancer cells (CM) and surgical wound fluids from patients who underwent IORT (RT-WF) and from patients after breast-conserving surgery alone (WF). We incubated two breast cancer cell lines (MCF-7 and MDA-MB-468) with WF, RT-WF, CM or WF + CM and measured radiobiological response of cells. We measured the level of double-strand breaks, induction of apoptosis and the changes in expression of genes related to DNA damage repair. We observed that stimulation with RT-WF and with WF + CM-induced double-strand breaks and increased expression of DNA damage repair-related genes, which was not observed after stimulation with WF. These results suggest that IOERT induces secretion of bystander factors mediating the genotoxic effect of ionizing radiation.

## Introduction

Breast cancer is the most common cancer in women and the second most common cancer in the world [1]. Although breast-conserving surgery can often be an effective course of treatment for early-stage breast cancer, the tumour cells remaining after excision can proliferate and form metastasis and recurrences, which are the leading cause of breast cancer-related deaths [2]. Irradiation of the entire breast and the positive lymph nodes after surgery was implemented to kill the residual cancer cells and reduce the risk of locoregional and distant recurrence, and breast cancer-related deaths following treatment [2]. Published data suggest that most of the cancer cell foci remaining after tumour excision are located up to 1 cm from the edge of the index tumour [3]. Irradiation of this area is the basis of intraoperative radiation therapy (IORT), a technique in which radiation is delivered in a single, high-dose fraction, directly to the tumour bed during surgery [4]. Treatment with IORT yields similar results in terms of survival and healthy tissue toxicity as the whole breast radiation therapy, with the benefit of reduced treatment time [5]. Apart from the delivery as a sole radiation treatment, IORT may also be delivered as a high-dose boost followed by the administration of fractionated whole breast irradiation, which nullifies the delay time between surgery and radiation therapy [6]. Although IORT exerts its effect mainly through the killing of cancer cells present in the irradiated volume, there are reports suggesting that it may also act by altering the environment of irradiated tumour bed.

The tumour bed presents an interesting target for preventing the development of local recurrences, not only because of the presence of a high number of cancer cells, but also because the wound healing response initiated by the surgical procedure changes the microenvironment, making it more favourable for the growth of cancer cells [7]. Although the wound healing response alters the tumour recurrence rate, administration of adjuvant radiotherapy also significantly impacts the microenvironment [8]. Accordingly, Belletti and colleagues [9] found that administration of IORT at a dose of 20 Gy changes the molecular composition of wound fluid and abrogates its stimulatory effect on migration, invasion and proliferation of breast cancer cells. Although the impact of IORT on wound fluids has already been described, the mechanism underlying this change has yet to be uncovered.

There is considerable evidence suggesting that ionizing radiation affects not only the cells targeted directly but also expands its effects to the non-irradiated neighbouring cells. This phenomenon is called radiation-induced bystander effect (RIBE), and it has been shown to induce end points resembling radiation response in the non-irradiated cells, like DNA damage, genetic instability, malignant transformation and cell death [10–13]. Induction of these end points is mediated through gap junction communication and soluble factors secreted by irradiated cells. Taking into consideration the changes observed in the composition and biological activity of surgical wound fluids following the administration of IORT, we hypothesized that this phenomenon might in part be caused by RIBE-related factors, secreted by cells irradiated during IORT. To investigate the difference caused by IORT in wound fluid activity, we incubated two breast cancer cell lines (MCF-7 and MDA-MB-468) with surgical wound fluids from patients after quadrantectomy (WF) and after quadrantectomy followed by boost IORT (RT-WF). In order to determine whether the change in RT-WF activity is caused by factors secreted by the irradiated cells, we collected conditioned medium from culture of irradiated breast cancer cells (CM) and incubated breast cancer cells with CM, and with WF in combination with CM. We investigated the common markers of response to irradiation and to RIBE: level of double-strand breaks, cellular apoptosis and expression of DNA damage repair pathway-related genes. The results of this paper suggest that wound fluids collected after IORT treatment induce radiobiological response in breast cancer cells. The response of cells stimulated with RT-WF was similar to the response of cells stimulated with combination of WF and CM, implying a similar mode of action.

## Materials and methods

### Wound fluid collection

Surgical wound fluids were collected from 16 female patients, treated for breast cancer in Greater Poland Cancer Centre in Poznan, Poland, with patients consent. The wound fluids were collected from the group of patients in which 80% of the treated malignancies were ER+/PR+ and HER2-, the tumours were no larger than 2.5 cm in diameter, and no metastasis was present. Patients were assigned to two groups, each group consisting of 8 patients. In the first group (WF), the patients underwent breast-conserving tumour resection (quadrantectomy). In the second group (RT-WF), the patients received intraoperative electron radiotherapy (IOERT) with a dose of 10 Gy after quadrantectomy. Wound fluids were drained up to 48 h after the surgery, centrifuged for 30 min at 300×*g* in 4 °C, sterile-filtered and stored at − 80 °C.

### Cell culture

The MCF-7 (ER positive, PR positive, HER2 negative) and the MDA-MB-468 (ER negative, PR negative, HER2 negative) cell lines were obtained from American Type Culture Collection (ATCC). Cells were cultured in a humidified atmosphere with 5% carbon dioxide in air at 37 °C. Both cell lines were cultured in Dulbecco modified Eagle medium (Biowest, France) supplemented with 10% foetal bovine serum (Biowest, France) and 1% penicillin/streptomycin 10,000 U/ml (Merck Millipore, Germany). The MCF-7 cells were additionally supplemented with 0.01 mg/ml insulin (Bioton, Poland).

### Conditioned medium collection

Conditioned medium (CM) was collected from irradiated MCF-7 and from irradiated MDA-MB-468 cells. Cells were irradiated in suspension with a dose of 10 Gy administered at approximately 2.5 Gy/min using GammaCell® 1000 Elite (BestTheratronics Ltd, Canada) with Caesium-137 source. After irradiation cells were cultured for 24 h after which CM was collected, sterile-filtered and stored at − 80 °C. For the stimulation of breast cancer cells, the CM of matching donor cell line was chosen.

### Cell treatment

The two cell lines were treated with wound fluids and conditioned medium in four variants: 10% CM in DMEM with 10% FBS (CM); 10% WF in DMEM without FBS (WF); 10% RT-WF in DMEM without FBS (RT-WF); 5% CM and 5% WF in DMEM without FBS (WF + CM). Cells were stimulated for the time indicated in the following sections.

### Flow cytometry

Cells were stimulated with wound fluids and conditioned medium and analysed at 9 time points: 30 min and 1, 2, 4, 8, 24, 48, 72 and 96 h after addition of fluids. Cells were then collected using Accutase (Biowest, France), fixated with BD Cytofix/Cytoperm™ Fixation/Permeabilization Solution (BD Biosciences, NJ, USA) and stained with fluorochrome-conjugated monoclonal antibodies: anti-human active caspase-3 antibody (Alexa Fluor 647 conjugated, rabbit IgG) (BD Biosciences, NJ, USA, Catalogue No. 552933), anti-human cleaved PARP antibody (PE conjugated, mouse IgG1) (BD Biosciences, NJ, USA Catalogue no. 552933) and anti-human γH2AX antibody (Alexa Fluor 488 conjugated, mouse IgG1) (BD Biosciences, NJ, USA Catalogue No. 560445). The stained cells were analysed using BD Accuri C6 (BD Biosciences, NJ, and USA). For quantification of each fluorescence signal, the median fluorescence intensity (MFI) was used. The results were normalized to the MFI of control (untreated) cells for each time point analysed.

### RNA isolation and RT-qPCR

Cells were stimulated with wound fluids and conditioned medium for 24 h. After that time, cells were collected and RNA was isolated using TRI Reagent^®^ (Sigma-Aldrich, MO, USA) according to manufacturer’s instructions. The first-strand cDNA was synthesized using 1 µg of RNA as a template, with iScript™ RT-qPCR cDNA Synthesis Kit (Bio-Rad, CA, USA), according to manufacturer’s instructions. RT-qPCR was carried out using FastStart Essential DNA Probes Master reaction mix (Roche, Germany), Universal ProbeLibrary hybridizing probes (Roche, Germany) and specific primers (Sigma-Aldrich, MO, USA). The list of primer sequences used in this study is provided in Table 1. The results were presented as a relative mRNA expression level calculated with the 2^−ΔΔCT^ method, using β-2 Microglobulin as a reference gene.

**Table 1:** Sequences of forward and reverse primers used for RT-qPCR

Gene	Forward primer	Reverse primer
*BRCA2*	CCTGATGCCTGTACACCTCTT	GCAGGCCGAGTACTGTTAGC
*MSH2*	GAGCCCTTAACCTTTTTCAGG	TTGTCCTTGAGGGGTTTTACAC
*MSH6*	AATGACATTCTAATAGGCTGTGAGG	AACCCATCTGGGCCATTAC
*RAD51*	GCAAGCGAGTAGAGAAGTGGA	TGCATCTGCATTGCCATTA
*XPA*	CGAGTATCGAGCGGAAGC	TTACATTAGCCATGCCTCCA
*XRCC1*	CTGGGACCGGGTCAAAAT	CAAGCCAAAGGGGGAGTC
*XRCC4*	TGGTGAACTGAGAAAAGCATTG	TGAAGGAACCAAGTCTGAATGA

### Ethical approval

The research has been conducted in compliance with the principles of Good Clinical Practice and Declaration of Helsinki, the collection of wound fluids was approved by the Bioethics Committee of Poznan University of Medical Sciences, Study Number 756/16. The written consent has been obtained from all the patients taking part in the research.

### Statistical analysis

For the statistical analysis, the one-way ANOVA test with Tukey’s post hoc test was performed. The differences were considered statistically significant at *p* < 0.05—*; *p* ≤ 0.01—**; *p* ≤ 0.001—***; *p* ≤ 0.0001—****. The tests were performed using GraphPad Prism version 6.01 (GraphPad Software, CA, USA).

## Results

### IORT changes the level of wound fluid-induced DNA double-strand breaks in breast cancer cells

**Fig. 1:**
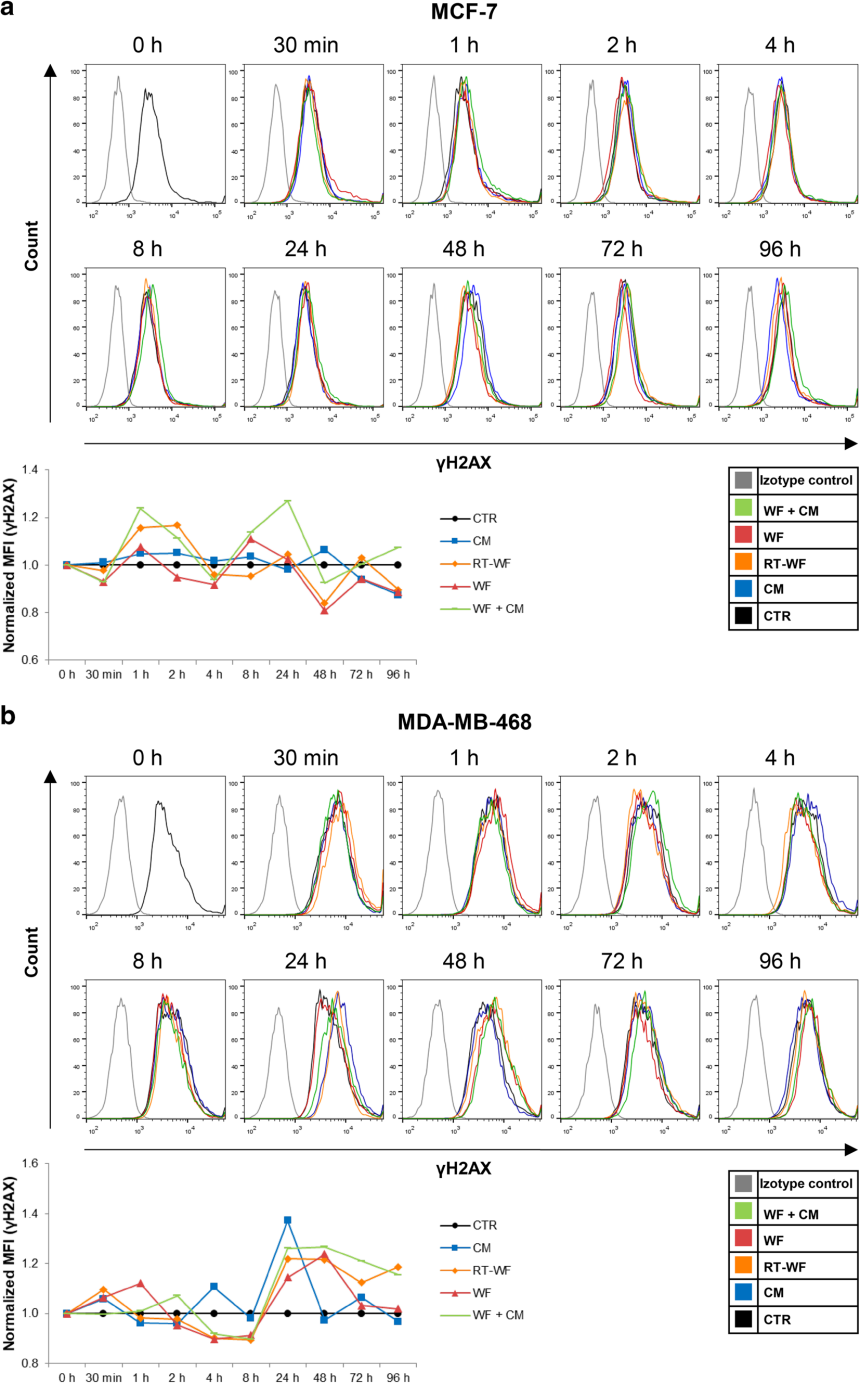
IORT alters the level of wound fluid-induced double-strand breaks (DSB) in breast cancer cells. Figure presents time course of the γH2AX level changes measured by flow cytometry in the **a** MCF-7 cells and **b** MDA-MB-468 cells. Data are presented as histograms from representative samples and as graphs of mean fluorescence intensity (MFI) normalized to the untreated control (mean of n experiments ± standard deviation). *N* = at least 3 independent biological replicates. *CM* conditioned medium collected from irradiated cells, *RT-WF* cells stimulated with 10% wound fluid collected after surgery and intraoperative radiotherapy, *WF* cells stimulated with 10% wound fluid collected after surgical excision, *WF* + *CM* cells stimulated with 5% conditioned medium and 5% surgical wound fluid

The induction of DNA double-strand breaks (DSBs), marked by the γH2AX increase, is a well-described effect of the medium-mediated RIBE [10]. Using flow cytometry, we measured the level of γH2AX in the breast cancer cells after stimulation with wound fluids and CM in four variants—CM/WF/RT-WF/WF + CM at different time points. In the MCF-7 cell line, we observed an increase in γH2AX level in cells treated for 1 and 2 h with RT-WF and for 1, 2, 8 and 24 h with WF + CM; however, the change was not statistically significant (Fig. 1a). The MDA-MB-468 cells stimulated for 24 and 48 h with RT-WF, WF and with WF + CM showed a similar increase in γH2AX level (Fig. 1b). The increase in DSB level was observable earlier in the MCF-7 cell line than in the MDA-MB-468 cell line, however, in both cases stimulation with RT-WF and with WF + CM-induced DNA damage. Although the level of DSBs varied significantly after administration of different wound fluids from one group, these results might imply a role of RIBE in wound fluid effects on breast cancer cells.

### IORT increases wound fluid-induced apoptosis in triple-negative breast cancer cells

**Fig. 2:**
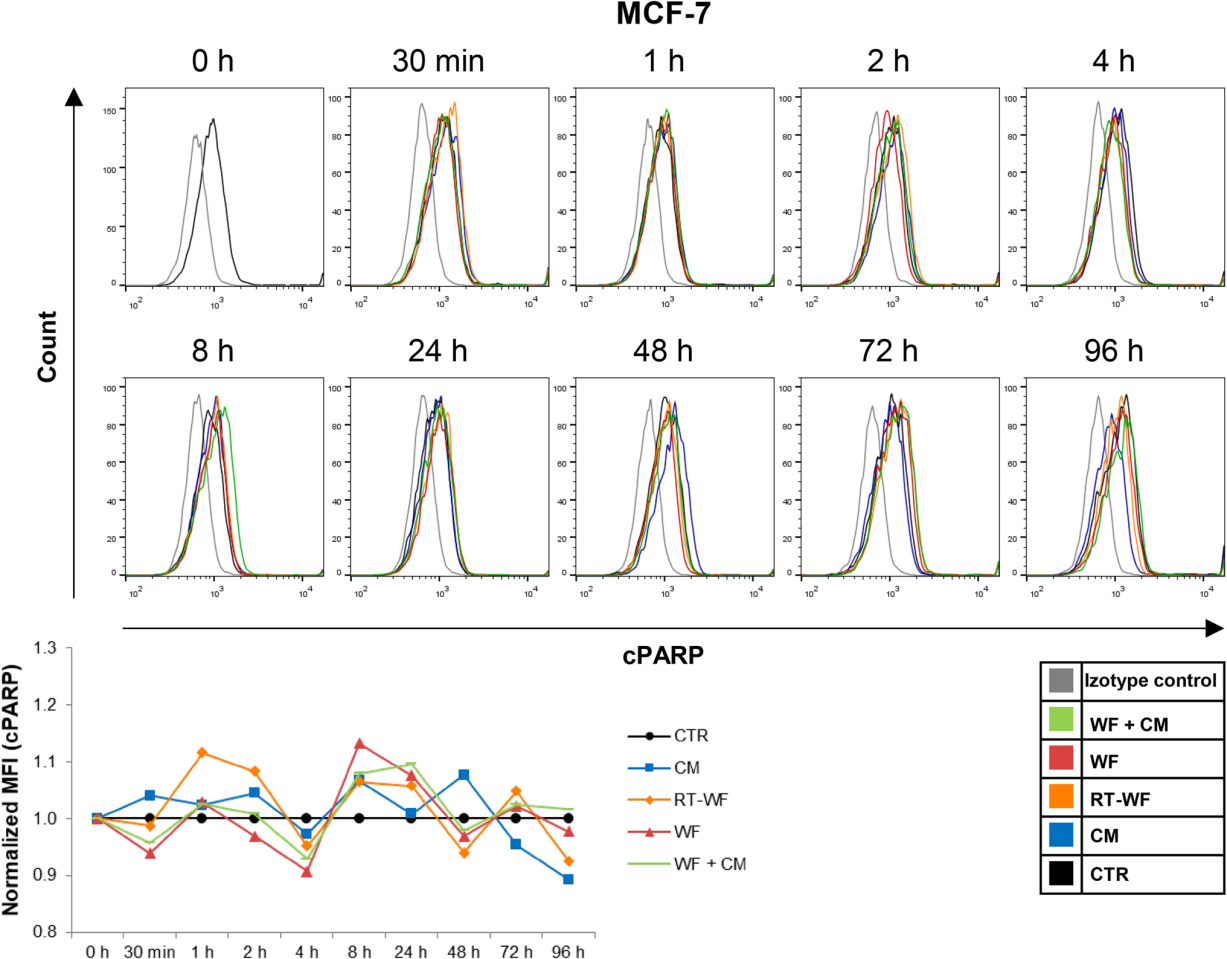
IORT does not impact the wound fluid-induced apoptosis in the MCF-7 cell line. Time course of the cleaved PARP (cPARP) level measured by flow cytometry in the MCF-7 cells. Data are presented as histograms compiled from samples representative of each group and as means ± standard deviation, normalized to the untreated controls. *N* = at least 3 independent biological replicates. *CM* conditioned medium collected from irradiated cells, *RT-WF* cells stimulated with 10% wound fluid collected after surgery and intraoperative radiotherapy, *WF* cells stimulated with 10% wound fluid collected after surgical excision, *WF* + *CM* cells stimulated with 5% conditioned medium and 5% surgical wound fluid

**Fig. 3:**
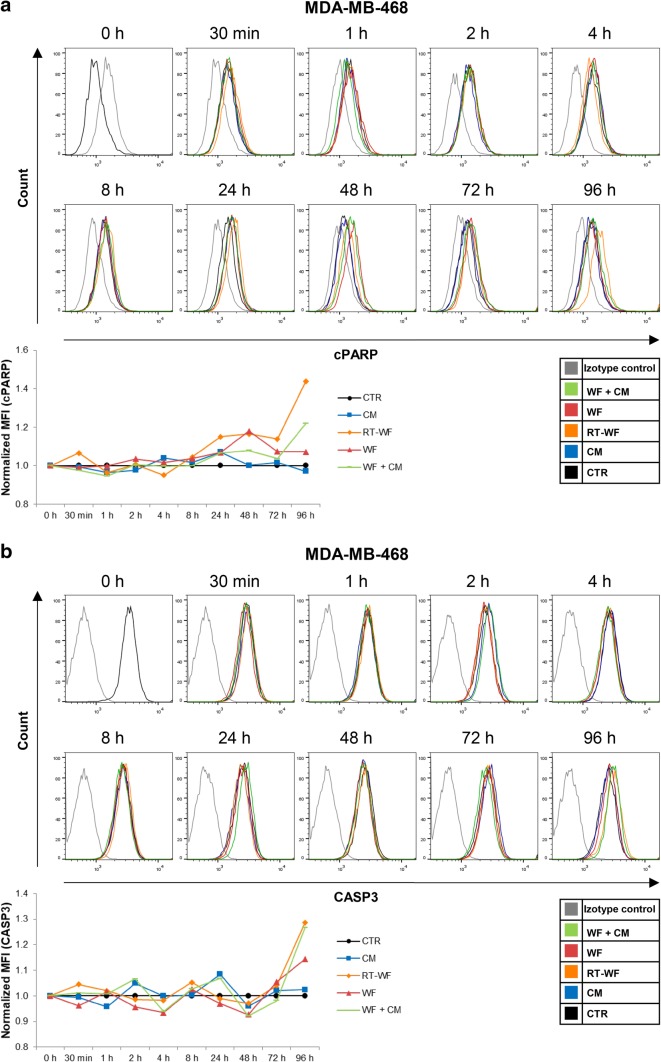
IORT alters the induction of apoptosis caused by the prolonged wound fluid stimulation in the MDA-MB-468 cells. Time course of the **a** cleaved PARP (cPARP) level and **b** active caspase 3 (CASP3) level measured by flow cytometry in the MDA-MB-468 cells. Data are presented as histograms compiled from samples representative of each group and as means ± standard deviation, normalized to the untreated controls. *N* = at least 3 independent biological replicates. *CM* conditioned medium collected from irradiated cells, *RT-WF* cells stimulated with 10% wound fluid collected after surgery and intraoperative radiotherapy, *WF* cells stimulated with 10% wound fluid collected after surgical excision, *WF* + *CM* cells stimulated with 5% conditioned medium and 5% surgical wound fluid

Induction of apoptosis has long been assumed as part of a response to RIBE [13]. After we analysed the changes in the DSB levels, we decided to investigate, whether the wound fluids and CM might induce cell death through apoptosis. To assess the activation of apoptosis in breast cancer cells, we measured the expression of two markers specific for apoptosis—the active caspase 3 (CASP3) and cleaved poly (ADP-ribose) polymerase (cPARP), by flow cytometry. There is evidence showing that the MCF-7 cell line has a functional deletion in CASP3 gene; however, the cell line retains the capacity to undergo apoptotic death [14], which is why for this cell line we chose to measure the cPARP level only. Analysis was performed on breast cancer cells incubated with CM, RT-WF, WF or WF + CM. Stimulation of MCF-7 cells did not result in significant changes in cPARP level compared to the level in control cells (Fig. 2). In the MDA-MB-468 cell line, the stimulation with RT-WF for 24 h or longer increased the level of cPARP (Fig. 3a). Stimulation for 96 h with RT-WF and WF + CM also increased the expression of CASP3 in this cell line, although these changes were not statistically significant (Fig. 3b). These results suggest that soluble factors present in RT-WF might induce apoptosis in a cell type-specific manner.

### Wound fluids induce DNA damage repair pathways in breast cancer cell lines

**Fig. 4:**
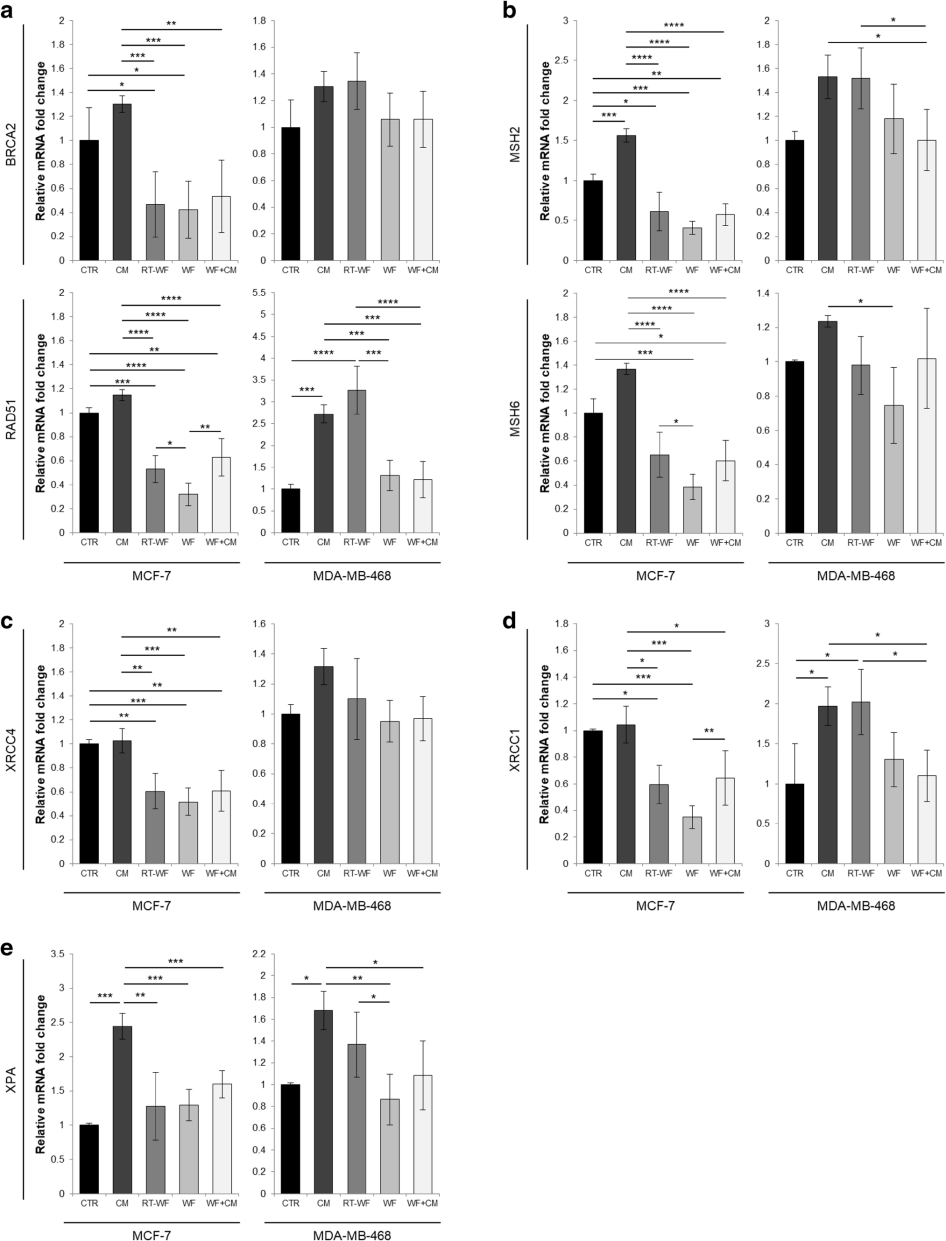
IORT modifies the effect of wound fluids on the activation of the DNA damage repair-related pathways in the breast cancer cells, marked by the up-regulation of the DNA repair-related genes. RT-qPCR was used to measure the expression of chosen DNA damage repair pathway genes: **a** homologous recombination (genes: RAD51, BRCA2), **b** mismatch repair (genes: MSH2, MSH6), **c** non-homologous end joining (gene: XRCC4), **d** base excision repair (gene: XRCC1), **e** nucleotide excision repair (gene: XPA). Data are presented as histograms compiled from samples representative of each group and as means ± standard deviation, normalized to the untreated controls. N = at least 3 independent biological replicates. *CM* conditioned medium collected from irradiated breast cancer cells, *RT-WF* wound fluid collected 48 h after surgical excision of the tumour followed by IORT treatment, *WF* wound fluid collected 48 h after surgical excision of the tumour, *WF* + *CM* combination of equal volumes of wound fluid collected after surgical excision of the tumour with conditioned medium from irradiated breast cancer cells. Results were considered statistically significant at *p < 0.05; **p ≤ 0.01; ***p ≤ 0.001; ****p ≤ 0.0001

It has been proven that signals secreted by irradiated cells induce DNA damage in non-irradiated bystander cells [10]. To gain further insight into the types of DNA damage response that might be induced in breast cancer cells by wound fluids, we decided to investigate the changes in expression of genes related to the DNA damage repair mechanisms. Figure 4 presents collected results of the gene expression analysis performed on breast cancer cells stimulated for 24 h with wound fluids. First we investigated the expression of genes associated with homologous recombination (HR)—BRCA2 and RAD51 (Fig. 4a). Incubation of the MCF-7 cells with wound fluids resulted in an overall down-regulated expression of HR-related genes, which was not observed in the CM-stimulated cells. In comparison with WF-stimulated cells, the cells incubated with RT-WF and with WF + CM showed higher expression of RAD51. In the MDA-MB-468 cell line, the 24-h stimulation with CM and with RT-WF resulted in an increase in RAD51 expression, together with a statistically insignificant increase in BRCA2 expression, in comparison with control cells.

Next we investigated mismatch repair (MMR). For this analysis, we chose two genes—MSH2 and MSH6 (Fig. 4b). In the MCF-7 cell line, we have observed decreased expression of both MSH2 and MSH6 after stimulation with RT-WF, WF and with WF + CM, in comparison with control cells. Stimulation of MCF-7 cells with CM resulted in increased expression of MMR-related genes.

Consequently we investigated the changes in expression of XRCC4, which is associated with non-homologous end joining (NHEJ) repair (Fig. 4c). The expression of XRCC4 gene in MCF-7 cells stimulated with RT-WF, WF and with WF + CM was significantly lower than in control cells or in the cells stimulated with CM.

For the evaluation of the base excision repair (BER) mechanism activation, we chose to measure changes in XRCC1 expression (Fig. 4d). Similar to the XRCC4 expression, stimulation of MCF-7 cells with RT-WF, WF and WF + CM resulted in down-regulation of XRCC1 compared to control or CM stimulated cells. In the cells stimulated with RT-WF and WF + CM, we observed higher expression of XRCC1 than in cells stimulated with WF. In the MDA-MB-468 cell line, stimulation with CM and with RT-WF resulted in higher expression of BER-related gene than control cells.

To test the changes in nucleotide excision repair (NER) pathway activation, we measured the expression of XPA gene (Fig. 4e). In both MCF-7 and MDA-MB-468 cells stimulation with CM resulted in a significant increase in XPA expression. Stimulation of MDA-MB-468 cells with RT-WF resulted in higher XPA expression than stimulation with WF.

Analysis of gene expression revealed that 24-h stimulation with CM can activate various DNA damage repair pathways in breast cancer cells and that this effect is dependent on the cell line. We have also observed that the cells stimulated with RT-WF showed higher expression of genes involved in HR, MMR, NHEJ and NER than cells stimulated with WF, and this effect also depended on the cell line used.

## Discussion

Local relapse is observed in a significant fraction of breast cancer patients [15]. The procedure of surgical excision itself perturbs the local microenvironment of the tumour through the activation of wound healing processes and inflammation, which are likely to contribute to the increased risk of recurrence [16, 17]. The surgical wound fluid, which accumulates in the lumpectomy cavity, is formed by inflammatory exudates and has been shown to stimulate growth, motility and invasiveness of breast cancer cells *in vitro* [9, 18, 19]. Research conducted by Belletti *et al*. [9] proved that the administration of intraoperative radiotherapy (IORT) changes the molecular composition of the surgical wound fluid, impairing its stimulatory effect on the growth of breast cancer cells. Furthermore, wound fluids were shown to stimulate stem-like phenotype of breast cancer cell lines; however, the effect seems to be highly dependent on the histological subtype of the line [20, 21]. Some authors also observed that this stimulatory effect was also impaired by IORT, which is consistent with the results presented by Belletti et al. [9]. All the aforementioned results support the theory that the surgical intervention induces wound healing processes, modifying the growth of tumour cells and increasing the risk of recurrence.

There are several reports indicating that the stimulatory effect of surgical wound fluids (WF) on breast cancer cells is impaired by administration of IORT; however, the mechanism of this change in biological activity is not well described. Research shows that after administration of ionizing radiation the effects are observable not only in the cells irradiated directly, but are also mediated through direct cell-to-cell contact and soluble factors to the non-irradiated bystander cells. We hypothesized that the IORT-induced change in biological activity of the surgical wound fluids might in part be caused by the molecules mediating radiation-induced bystander effect (RIBE), produced by the irradiated breast cancer cells present in the tumour bed. The radiation-induced bystander effect has been linked with the induction of various genotoxic effects in the non-irradiated cells [22–26], and the effects of RIBE are observed for doses up to 10 Gy, suggesting that this genotoxic effect might be relevant to doses used during IORT administration [27]. To investigate the difference induced by administration of intraoperative electron radiotherapy (IOERT) in wound fluids and its underlying mechanism, we collected the conditioned medium from the culture of breast cancer cell lines irradiated *in vitro* (CM), and surgical wound fluids from two groups of patients: patients who underwent quadrantectomy without additional IOERT (WF) and patients treated with 10 Gy boost IOERT (RT-WF). The wound fluids were drained for 48 h after surgery, and the conditioned medium was collected 24 h after irradiation. We measured RIBE-related effects in two breast cancer cell lines—MCF-7 and MDA-MB-468—after incubation with CM, WF, RT-WF and a combination of WF and CM. We hypothesized that if RIBE plays a significant role in the activity of RT-WF, the addition of CM containing the bystander factors to the WF should change its biological activity, making it similar to that of RT-WF.

Since the induction of DNA double-strand breaks is considered one of the most important genotoxic effects of ionizing radiation and has been observed as the effect of RIBE, we decided to investigate the level of DSBs in breast cancer cell lines stimulated with wound fluids by cytometric measurement of the γH2AX histone level in cells. Stimulation of breast cancer cell lines with RT-WF and with WF + CM increased the measured level of DSBs. This suggests not only that the administration of IOERT induces genotoxic activity in surgical wound fluids, but also that the mechanism behind the induction might be related to RIBE. Our results find confirmation in the literature. Han et al. proved that irradiation of skin fibroblasts with low doses of α-particle radiation induces DSB formation in the non-irradiated bystander cells [28]. Mothersill et al. [29] also proved that medium from irradiated cells (human keratinocytes) initiates the apoptotic cell death 48 h after the conditioned medium transfer [13]. Since apoptosis is one of the cell death pathways induced by both the direct radiation and by RIBE [30], we decided to investigate the level of apoptosis in breast cancer cell lines stimulated with wound fluids using cytometric measurement of the level of two apoptotic proteins: caspase 3 and cleaved PARP. In this study, we observed that only the prolonged stimulation (96 h) of the triple-negative breast cancer cell line with RT-WF resulted in increased expression of apoptotic proteins indicative of apoptosis activation. The results previously published by our group show that a 4-day stimulation with RT-WF induces the extrinsic apoptotic pathway in breast cancer cells, measured at the transcript level [31]. Jella and colleagues proved that 72-h incubation of cells with conditioned medium from irradiated cells induces cellular death through apoptosis and mitotic catastrophe [25]. Induction of DSBs and cell death by RT-WF suggests that IOERT induces RIBE-like properties in surgical wound fluid.

Researchers show that genotoxic effect of RIBE is dependent on production of reactive oxygen species (ROS), among other soluble toxic factors [10]. Chen et al. [26] confirmed that RIBE mediated through secreted factors relies on the mitochondria-dependent signalling, which causes an up-regulation of ROS production in the bystander cells. The oxidative stress-inducing factors present in the conditioned medium from irradiated cells were shown to be stable for at least 24 h and were still active after consecutive freezing and thawing [32]. ROS induces various DNA-damaging effects, such as base modifications, DNA crosslinks and single- and double-strand breaks, and for the cell to repair the oxidative DNA damage a variety of repair pathways has to be activated [33, 34]. Since our results indicate a role of RIBE, which is dependent on oxidative stress and ROS production, we investigated the activity of DNA damage repair pathways related to oxidative stress response in cells stimulated with wound fluids and conditioned medium, by measuring the transcript level of DNA damage repair-related genes. We found that the expression of genes related to DNA repair is significantly increased after stimulation with RT-WF in comparison with stimulation with WF. In the ER+/PR+ breast cancer cell line (MCF-7), we have observed that stimulation with RT-WF and the WF + CM resulted in higher expression of genes related to HR, BER and MMR pathways, than stimulation with WF alone. In the triple negative breast cancer cell line (MDA-MB-468) stimulated with RT-WF, we have observed a significant increase in the expression of genes related to HR, MMR and NER, and a nonsignificant increase in the expression of the BER pathway gene. In both cell lines, the HR- and NER-related genes were up-regulated after stimulation with CM only. The DNA damage repair through the HR, BER, MMR and NER pathways has all been shown to take part in DNA damage response after the appearance of the oxidative DNA damage [35]. Some of the investigated repair pathways were also up-regulated after stimulation with WF + CM in comparison with WF, which suggests that the difference between the biological activity of RT-WF and WF may in part be caused by the presence of factors present in the CM. Although incubation of the MCF-7 cells with WF, RT-WF and WF + CM resulted in an overall down-regulated expression of the DNA damage repair genes (genes related to HR, NHEJ, BER, MMR), we observed significant differences between the RT-WF, WF and WF + CM stimulation. Similar down-regulation of the DNA damage-repair genes after stimulation with wound fluids was not observed in the MDA-MB-468 cells. This may indicate that the response of breast cancer cells to the factors present in surgical wound fluids depends on the differences between chosen cell lines. Similar differences in cellular response were also observed by other authors [9, 21]. Since our results implicate a radiation-related response of the cells stimulated with RT-WF and with WF + CM, the different response of the two breast cancer cell lines might be caused by the intrinsic radiation sensitivity of the chosen cell lines. It has been proven that in some tumours, cancer stem cells show higher resistance to ionizing irradiation-induced cell killing than their progeny [36, 37]. In breast cancer stem cells, this effect is caused, at least partially, by the increased efficiency of the free radical scavenging systems [36]. The MDA-MB-468 cell line has a larger population of cells presenting stem-like phenotype than the MCF-7 cell line, which suggests it might have more effective DNA damage repair mechanisms [21, 38]. While our group observed significant changes in the activation of DNA damage repair pathways, these changes did not correlate directly with the measured levels of apoptosis. The differences in apoptosis induction in stimulated cells were much less pronounced than the differences in the expression of DNA damage repair genes. This observation in cells stimulated with RT-WF and with WF + CM might in part be caused by a sufficient activation of the repair systems for the cell to avoid death through apoptosis.

## Conclusions

This is the first research investigating the potential connection between the radiation-induced bystander effect and the anti-cancer properties of wound fluids accumulating in the surgical cavity after administration of intraoperative radiotherapy. We observed that stimulation of breast cancer cells with RT-WF and with WF + CM resulted in higher level of double-strand breaks and increased expression of DNA damage repair-related genes in comparison with cells stimulated with WF, which suggests that RT-WF induces radiation-like damage in breast cancer cells. Since the changes we observed were induced after the incubation with RT-WF and WF + CM, but not after the incubation with WF, we conclude that IOERT induces a release of bystander factors, which mediate the genotoxic effect of radiation. The tumoricidal effect of RT-WF described here might be one of the factors contributing to the decreased recurrence rates after IOERT [39].
